# Patient-reported 1-year outcome not affected by body mass index in 3,327 total knee arthroplasty patients

**DOI:** 10.1080/17453674.2019.1604940

**Published:** 2019-04-17

**Authors:** Anders Overgaard, Lars Lidgren, Martin Sundberg, Otto Robertsson, Annette W-Dahl

**Affiliations:** a The Parker Institute, Copenhagen University Hospital Fredriksberg, Copenhagen, Denmark;; b The Swedish Knee Arthroplasty Register, Lund, Sweden;; c Lund University, Faculty of Medicine, Department of Clinical Sciences Lund, Orthopaedics, Lund, Sweden

## Abstract

Background and purpose — Patient-reported outcome (PRO) in total knee arthroplasty (TKA) patients with high body mass index (BMI) is controversial. We compared pain, function, quality of life, general health, and satisfaction among different BMI categories preoperatively and 1 year after primary TKA.

Patients and methods — 4,318 patients were operated with a TKA for knee osteoarthritis in the Region of Skane in 2013–2015. In all, 3,327 patients (77%) had complete PRO data and information on BMI and were included. Preoperatively the patients filled in the Knee injury and Osteoarthritis Outcome Score (KOOS) and EQ-VAS (general health). 1 year postoperatively the same questionnaires were filled in together with a question asking whether they were satisfied with the surgery. Information on age, sex, BMI, and ASA grade were obtained from the Swedish Knee Arthroplasty Register. Each patient was classified as Outcome Measures in Rheumatology– Osteoarthritis Research Society International (OMERACT–OARSI) responder or not based on a combination of absolute and relative changes in scores. Welch’s t-test and a chi-square test were used in the statistical analysis.

Results — Both preoperatively and 1 year postoperatively the obese patients reported somewhat worse scores than the normal weight and overweight. The differences were small with 1 exception, the KOOS sport- and recreation function postoperatively, where normal-weight and overweight patients reported fewer problems than obese patients with a BMI over 35 (40 and 39 points vs. 31 points, p < 0.001). Similar proportions of patients were satisfied and categorized as OMERACT–OARSI responders in the different BMI categories.

Interpretation — The degree of improvement in PROs 1 year after TKA surgery does not seem to be affected by BMI.

As the number of primary knee arthroplasties, as well as the number of obese patients undergoing total knee arthroplasty (TKA), continues to increase, there has been more interest in the role of obesity as a risk factor for poor outcomes after TKA. In the literature, the influence of obesity on knee arthroplasty outcome diverges. Some studies show that obesity has no influence on TKA outcomes such as pain and function (Deshmukh et al. 2002, Stevens-Lapsley et al. 2010, Yeung et al. 2011, Baker et al. 2012, Collins et al. 2012), patient satisfaction (Yeung et al. 2011), early complications (Patel and Albrizio 2008, Yeung et al. 2011, Collins et al. 2012), and mid-term survival of the knee arthroplasty (Bourne et al. 2008, Bordini et al. 2009, Yeung et al. 2011, Collins et al. 2012). However, others have found worse outcomes regarding pain and function (Mulhall et al. 2007, Järvenpää et al. 2012, Issa et al. 2013, Liljensoe et al. 2013), satisfaction (Järvenpää et al. 2012), complications (Yasunaga et al. 2009, Järvenpää et al. 2010, Issa et al. 2013) and an increased risk of infection (Namba et al. 2005).

If orthopedic surgeons hesitate to operate on obese patients, because of a suspected greater risk of worse outcome after arthroplasty surgery, this may lead to disparity in surgical treatment among the general population. Considering the rising prevalence of obesity, it is of importance to evaluate whether the knee arthroplasty surgery benefits the patients in order to help patients and surgeons decide on treatment.

A rising public health concern about the influence of the obesity epidemic on the healthcare system furthers a need to substantiate the effect of obesity on treatments (King et al. 2013). We compared pain, function, quality of life, and general health preoperatively and 1 year postoperatively, as well as improvement and satisfaction 1 year postoperatively in patients operated on with TKA for knee OA, and stratified our analyses to investigate the effects obesity had on patient-reported outcomes (PROs).

## Patients and method

The study population consisted of 4,318 TKA patients (5,065 knees) operated on for knee osteoarthritis (OA) between 2013 and 2015 in the most southern region of Sweden (Region Skane). Of the 4,318 patients, 318 had bilateral simultaneous TKA and 429 bilateral staged TKA during the study period. For patients having bilateral simultaneous TKA the right TKA was considered and for patients with staged surgery, the later surgery was considered. Further, we excluded patients who did not have both preoperative and 1-year postoperative PRO data and those who had died during the follow-up year. Of the 4,286 available patients 77% had complete PRO data and information on BMI (954 patients had not complete PRO data and in 5 patients BMI was missing). The patient characteristics and the preoperative PROs available for the 991 patients excluded or lost to follow-up were similar to those included without clinically relevant differences (Table 1).

**Table 1.** Preoperative demographics and preoperative PROs for patients included and excluded in the study and the TKA/OA patients in the SKAR 2013–2015. Values are mean (95% CI) unless otherwise specified

Variable	Included (n = 3,327)	Excluded^a^ (n = 991)	p-value	SKAR (n = 35,932)
Women, n (%)	1,912 (58)	593 (60)	0.2	20,389 (57)
Age	69 (69–70)	69 (69–70)	1.0	69 (69–69)
BMI	29 (29–29)	29 (29–29)	0.02	29 (29–29)
ASA ≥ 3, n (%)	513 (15)	186 (19)	0.01	6,035 (17)
KOOS		n = 509		
pain	41 (40–41)	38 (37–39)	< 0.001	
symptoms	48 (47–49)	45 (43–46)	< 0.001	
ADL	46 (46–47)	43 (42–44)	< 0.001	
sport/rec	12 (12–13)	11 (10–12)	0.07	
QoL	24 (23–24)	20 (19–22)	< 0.001	
EQ-VAS		n = 527		
	70 (69–70)	64 (6–66)	< 0.001	

a Excluded or lost to follow-up.

BMI = body mass index, ASA = American Society of Anesthesiologists, KOOS = Knee injury and Osteoarthritis Outcome Score, ADL = activity in daily life function, Sport/rec = sport and recreation function, QoL = quality of life, VAS = visual analogue scale.

Patient characteristics such as sex, age, BMI, and ASA classification were obtained from the Swedish Knee Arthroplasty Register (SKAR). BMI was categorized according to the WHO classification underweight (< 18.5), normal weight (18.5–24.9), overweight (25–29.9), obese I (30–34.9), obese II (35–39.9), and obese III (≥ 40).

Preoperatively the patients filled in the disease-specific Knee injury and Osteoarthritis Outcome Score (KOOS) (Roos et al. 1998) and the generic instrument EQ-VAS (general health) (EuroQol Group 1990). 1 year postoperatively the same questionnaires were sent to the patients together with a question as to whether they were satisfied with the surgery. The patients were informed of the planned 1-year follow-up, but no reminders were sent if they did not respond.

The KOOS consists of 42 questions and includes 5 subscales. Each question is allotted a score from 0 to 4. A normalized score (100 indicating no symptoms and 0 indicating extreme symptoms) is calculated for each subscale.

The patients reported their self-perceived general health using the EQ-VAS on a scale (0–100) from the best (100) to the worst imaginable health status (0) and their satisfaction with the arthroplasty surgery using a 0–100 scale (VAS) in which 0 was the highest imaginable degree of satisfaction and 100 was the worst imaginable degree of satisfaction. The satisfaction (VAS) score was categorized into 5 groups: very satisfied (0–20), satisfied (21–40), moderately satisfied (41–60), dissatisfied (61–80), and very dissatisfied (81–100).

The KOOS was converted to Western Ontario and McMaster Universities Osteoarthritis Index (WOMAC) to be able to classify each patient as an Outcome Measures in Rheumatology–Osteoarthritis Research Society International (OMERACT–OARSI) responder or not at 1 year based on a combination of absolute and relative changes in WOMAC pain, function, and total scores (Pham et al. 2004). The outcome at 1 year was categorized into responders (high and low) and non-responders according to these criteria (Pham et al. 2004).

### Statistics

PRO, age, and BMI are presented as mean value (SD) and/or 95% confidence interval (CI). Welch’s t-test was used for comparisons of the KOOS and the EQ-VAS between the different BMI categories considering unequal variances and unequal sample sizes with assumption of normal distribution. Due to few patients in the underweight and obese III groups, these were analyzed together with the normal weight and obese II groups respectively. For analysis of proportions of sex, ASA grade, and OMERACT–OARSI responder rate the chi-squared test was used for comparisons. A difference between the groups of ≥ 8 points in KOOS and ≥ 15 mm in EQ-VAS was considered a clinically relevant difference for statistically significant results (p < 0.05). Multiple linear regression analysis was used to evaluate the relationship between BMI and change in KOOS pain and ADL function preoperatively to 1 year postoperatively adjusted for age and sex. In a further analysis ASA grade (ASA I, ASA II, and ASA ≥ III) and preoperative KOOS pain and ADL function respectively were included in the model in addition to age and sex. Statistical analyses were carried out using Stata version 14 (StataCorp, College Station, TX, USA).

## Results

Of the 3,327 patients, 58% were women, the mean age was 69 years, mean BMI 29, and 15% were classified as ASA ≥ III (Table 1). 0.2% of the included patients were underweight, 19% normal weight, 45% overweight, 27% obese I, 7% obese II, and 1% obese III.

Both preoperatively and 1 year postoperatively the obese patients reported statistically significant worse KOOS scores than the normal weight and overweight in most of the subscales without clinically relevant differences. The only exception was in Sport/Rec function postoperatively were normal-weight and overweight patients reported better outcome then obese II–III patients (40 (CI 38–42) and 39 (CI 38–41) vs. 31 (CI 28–34)) (Table 2).

**Table 2.** Patient reported outcome preoperatively and 1 year postoperatively in the different BMI categories. Values are mean (SD) CI

	Normal weight (N)	Overweight (O)	Obese I (I)	Obese II + III (II+)	p-value N vs O	p-value N vs I	p-value N vs II+	p-value O vs I	p-value O vs II+	p-value I vs II+
Preoperatively										
KOOS										
pain	43 (15) 42–45	41 (15) 40–41	39 (15) 38–40	37 (15) 35–38	0.002	< 0.001	< 0.001	0.004	< 0.001	0.009
symptom	50 (19) 48–51	48 (18) 47–49	47 (17) 46–49	45 (17) 43–47	0.04	0.006	< 0.001	0.3	0.004	0.04
ADL	49 (16) 48–50	47 (15) 47–48	44 (15) 43–45	42 (16) 40–44	0.01	< 0.001	< 0.001	< 0.001	< 0.001	0.08
sport/rec	14 (15) 13–16	13 (14) 12–14	11 (14) 10–11	9 (14) 7–11	0.03	< 0.001	< 0.001	< 0.001	< 0.001	0.1
QoL	25 (14) 24–26	24 (14) 24–25	23 (14) 22–24	21 (13) 19–22	0.3	0.003	< 0.001	0.008	< 0.001	0.02
EQ–VAS	72 (21) 70–74	71 (21) 70–72	67 (21) 65–68	64 (22) 61–66	0.4	< 0.001	< 0.001	< 0.001	< 0.001	0.05
Postoperatively										
KOOS										
pain	80 (19) 79–82	80 (19) 79–81	78 (19) 77–79	78 (19) 77–79	0.4	0.02	0.02	0.06	0.07	0.6
symptom	77 (17) 76–78	76 (17) 75–77	75 (17) 74–76	75 (17) 74–76	0.3	< 0.001	0.1	0.06	0.4	0.8
ADL	81(18) 79–82	79 (19) 78–80	76 (19) 75–77	74 (20) 72–76	0.5	< 0.001	< 0.001	< 0.001	< 0.001	0.1
sport/rec	40 (27) 38–42	39 (27) 38–40	34 (27) 32–36	32 (28) 32–35	0.3	< 0.001	< 0.001	< 0.001	< 0.001	0.2
QoL	66 (23) 64–68	65 (24) 64–66	62 (24) 60–63	61 (25) 60–63	0.4	< 0.001	0.008	0.002	0.02	0.8
EQ–VAS	78 (19) 77–80	77 (19) 76–78	74 (20) 73–75	72 (20) 69–75	0.3	< 0.001	< 0.001	< 0.001	< 0.001	0.2
Change										
KOOS										
pain	32 (21) 30–34	34 (21) 33–35	35 (22) 33–36	36 (23) 34–39	0.08	0.02	0.006	0.3	0.07	0.2
symptom	27 (22) 25–29	28 (22) 27–29	27 (22) 26–29	28 (22) 27–29	0.3	0.7	0.05	0.5	0.1	0.5
ADL	31 (19) 30–33	32 (20) 31–33	32 (20) 31–34	32 (20) 31–33	0.8	0.3	0.6	0.4	0.6	0.9
sport/rec	26 (27) 24–28	26 (27) 25–28	23 (27) 21–25	23 (29) 19–26	0.8	0.05	0.09	0.006	0.04	0.7
QoL	41 (23) 39–43	41 (25) 40–42	39 (26) 38–41	41 (25) 38–44	0.8	0.1	0.8	0.1	1.0	0.3
EQ–VAS	5 (25) 3–7	5 (26) 4-–7	8 (26) 5–11	8 (25) 5–11	0.09	0.2	0.5	0.1	0.1	0.5

For abbreviations, see Table 1

**Figure 1.**
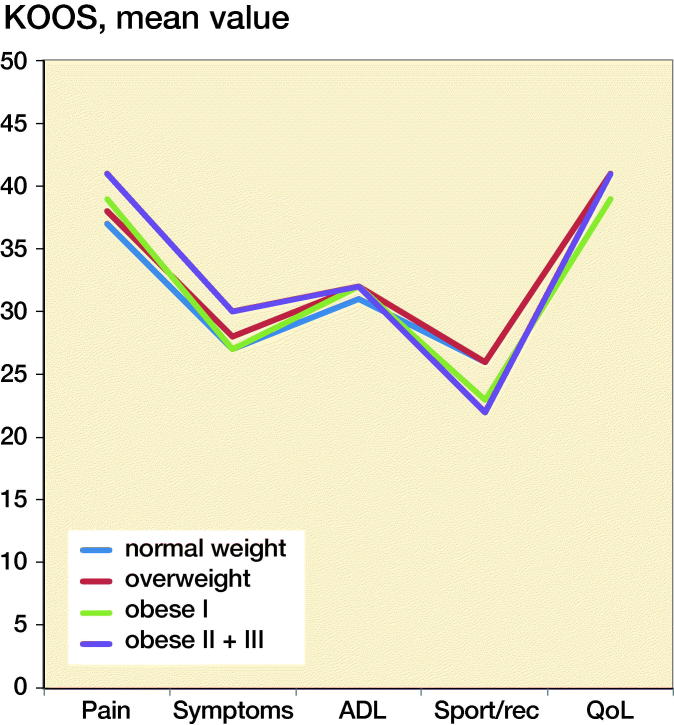
Mean changes preoperatively to 1 year postoperatively in KOOS 5 subscales in the different BMI categories.

The improvements were comparable in the KOOS subscales pain, symptoms, ADL function, and knee-related QoL without any clinically relevant differences. In Sport/Rec function the normal weight and overweight improved somewhat more than the obese patients although without any clinically relevant differences (Figure 1).

We could not show any effect of BMI on change in KOOS pain (0.1 [–0.05 to 0.3]) and ADL function (0.03 [–0.1 to 0.2]) when adjusting for age and sex. When we included ASA grade and preoperative KOOS pain and ADL function respectively in the models, BMI was not found to have any effect on change in KOOS pain but a statistically significant effect on change in ADL function (2 points less improvement/10 higher BMI units) (Table 3).

**Table 3.** Relationship between potential confounding factors to change in the KOOS pain and ADL function

Variable	Change in KOOS pain coefficient (CI)	p-value	Change in KOOS ADL coefficient (CI)	p-value
Age	0.1 (0.02–0.2)	0.02	–0.1 (–0.2 to –0.1)	0.02
Sex				
Male	Ref		Ref	
Female	–1.6 (–2.9 to –0.3)	0.02	–1 (–2.2 to –0.3)	0.1
BMI	–0.3 (–0.2 to 0.1)	0.7	–0.2 (–0.4 to –0.1)	0.004
ASA classification				
1	Ref		Ref	
2	–2.3 (–4 to –0.6)	0.008	–2.3 (–3.9 to –0.6)	0.008
3	–3.7 (–6.1 to –1.4)	0.002	–4.7 (–6.9 to –2.4)	< 0.001
Preoperative KOOS				
pain	–0.7 (–0.8 to –0.7)	< 0.001		
ADL	–0.6 (–0.7 to –0.6)	< 0.001		

For abbreviations, see Table 1.

**Figure 2.**
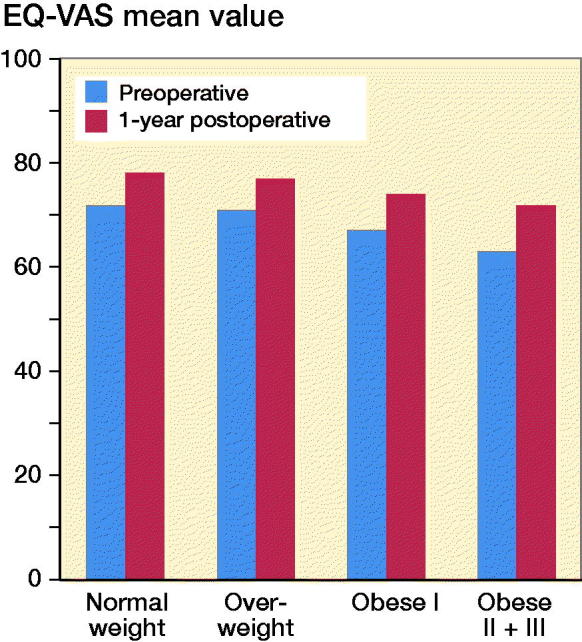
EQ-VAS (general health) mean value preoperatively and 1 year postoperatively in the different BMI categories.

The normal-weight and overweight patients reported somewhat better general health (EQ-VAS) preoperatively than the obese patients without statistically or clinically significant differences. The improvement in general health, among the different BMI categories, preoperatively to 1 year postoperatively was small: 5–8 points (Figure 2).

**Figure 3.**
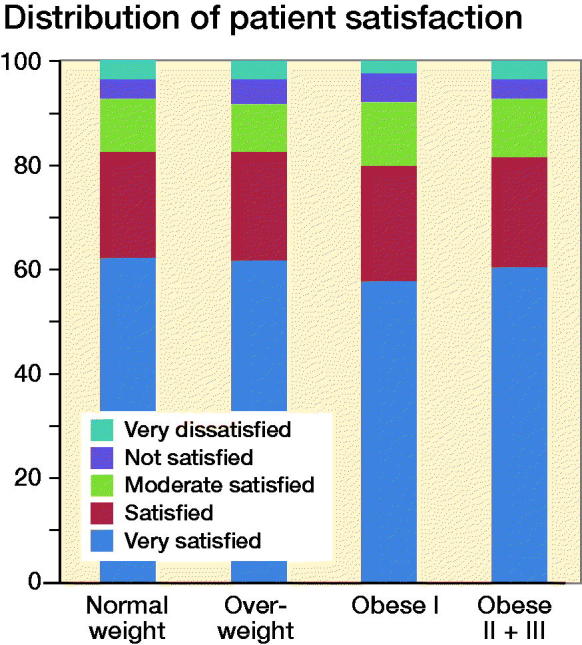
Patient satisfaction in the different BMI categories.

The proportion of satisfied patients varied between 80% and 83% in the different BMI categories (Figure 3).

**Figure 4.**
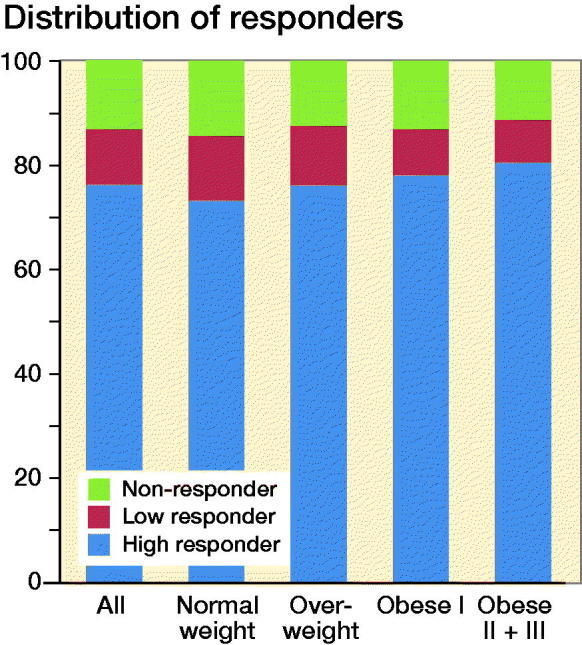
Responder classification in the different BMI categories.

Similar proportions (85–88%) of patients in the different BMI categories were classified as OMERACT–OARSI responders and the majority of the patients were classified as high responders: 73–80%. Patients with BMI ≥ 35 had the highest proportion of responders (Figure 4).

## Discussion

We found that obese primary TKA patients in southern Sweden reported similar knee-related pain, function, quality of life, and satisfaction as non-obese patients at 1 year after surgery, with a comparable proportion of OMERACT–OARSI responders. This may be valuable information for the knee arthroplasty surgeons when considering obese patients for TKA surgery.

Earlier studies have reported disadvantageous patient/surgeon-reported outcome in obese patients when compared with non-obese patients after TKA surgery (Järvenpää et al. 2012, Liljensoe et al. 2013, Issa et al. 2013) or similar results (Collins et al. 2012). However, the most recent studies from the United States and Western Europe are in line with our results (Daniilidis et al. 2016, Li et al. 2017, Giesinger et al. 2018). The reasons for dissimilarities may be differences in study size, loss to follow-up, time to follow-up, patient selection, and methods of scoring. The majority of these studies include relatively few patients and are from single centers (Järvenpää et al. 2012, Collins et al. 2012, Issa et al. 2013, Liljensoe et al. 2013, Daniilidis et al. 2016, Giesinger et al. 2018). Further, the follow-up time in the above-mentioned studies varies between 6 months and 11 years, which may influence the results. One of the strengths of our study is a large patient base that resembles the national data in Sweden, though gathered from only 5 centers.

In contrast to the other studies, our Swedish cohort included a relatively low proportion of obese patients (35%) and especially obese III patients (1%). The US FORCE-TJR cohort (2,964 TKA patients) included 53% obese patients, and 9% of these were obese III (Li et al. 2017), the UK cohort (402 TKA patients) included 51% obese patients of whom 7% were obese III (Giesinger et al. 2018), and the German cohort (199 TKA patients) consisted of 63% obese patients including 9% obese III (Daniilidis et al. 2016). The number of obese III patients is few in all studies except for the US FORCE-TJR cohort, which included 272 obese III patients.

Data support an increase in morbidity and mortality associated with severe obesity (Krushell and Fingeroth 2007, Vaishya et al. 2016) among patients receiving TKA, but for non-morbidly obese (overweight and obesity I–II) patients, the data are not clear.

The non-obese patients reported somewhat better general health than the obese patients but the differences were small (4–8) preoperatively with small changes 1 year after the TKA surgery in the different BMI categories. The small change in general health preoperatively to 1 year postoperatively indicate that the patients did not experience significant general health improvement from the TKA surgery and that a general health measure alone may not be a suitable outcome measure to evaluate the knee arthroplasty surgery.

We found no clinically relevant differences in the patient reported outcome between obese and non-obese patients measured by the KOOS at the 1-year follow-up after knee arthroplasty surgery except for the subscale sports and recreation function, which had a clinically and statistically significant different outcome when comparing obese II + III and normal weight and overweight patients. Severe obesity is associated with functional disability (Anandacoomarasamy et al. 2008). The subscale sport and recreation function includes more demanding functions such as squatting, running, jumping etc., which might explain the difference in this outcome measure.

Satisfaction has become a common measure to define a successful outcome when evaluating elective arthroplasty surgery. Though satisfaction does not always correlate with PRO-derived responder rates and it has been advocated that a poor outcome, as well as a good one, might be hidden when reporting the mean pain and function scores (Roos 2018). Our data show 80–82% of the patients in the different BMI categories were categorized to be “very satisfied” or “satisfied” with the surgery while 85–87% were classified as OMERACT–OARSI responders. Bourne et al. (2008) showed that the relationship between patient-reported outcome and patient satisfaction is multifactorial, which may to some extent explain the discrepancy in proportions between satisfied patients and OMERACT–OARSI responders in our study though our study clearly shows similar satisfaction rates among BMI categories.

Our patients had surgery in a region in the south of Sweden. These patients presented similar patient characteristics to patients operated on for OA with TKA in the whole country (Table 1). The patients excluded/lost to follow-up in our study for different reasons consisted of a somewhat higher proportion of women although without a statistically significant difference. However, we found a statistically significant difference in the mean BMI and proportion of ASA ≥ 3 patients but the difference was small (0.4% and 3% respectively) between the included patients and those excluded/lost to follow-up, though of no real clinical importance.

In 2015, almost 13,000 knee arthroplasties were performed in Sweden resulting in an age-standardized incidence of 132/100,000 inhabitants (SKAR 2016). That more than one-third of the patients having primary knee arthroplasty surgery for OA in Sweden 2015 were obese (BMI ≥ 30) may reflect on an increased risk of progression of OA in the obese population.

In summary, considering the similar patient-reported outcome in the different BMI categories, with reservations for the low number of obese III patients, BMI seems to have little effect on patient-reported outcome 1 year postoperatively in patients having a TKA for OA.

The study was conceived by OR, LL, MS, and AWD. AO, AWD, and OR performed the analyses. AO wrote the initial draft. All the authors contributed to the interpretation of the data and to a revision of the manuscript.

*Acta* thanks Hannu Miettinen for help with peer review of this study.
